# Orally administered heat-killed *Lactobacillus paracasei* MCC1849 enhances antigen-specific IgA secretion and induces follicular helper T cells in mice

**DOI:** 10.1371/journal.pone.0199018

**Published:** 2018-06-13

**Authors:** Satoshi Arai, Noriyuki Iwabuchi, Sachiko Takahashi, Jin-zhong Xiao, Fumiaki Abe, Satoshi Hachimura

**Affiliations:** 1 Food Ingredients and Technology Institute, Morinaga Milk Industry Co., Ltd., Zama-City, Kanagawa, Japan; 2 Next Generation Science Institute, Morinaga Milk Industry Co., Ltd., Zama-City, Kanagawa, Japan; 3 Research Center for Food Safety, Graduate School of Agricultural and Life Science, University of Tokyo, Bunkyo-ku, Tokyo, Japan; University of South Florida St Petersburg, UNITED STATES

## Abstract

Antigen-specific immunoglobulin (Ig) A plays a major role in host defense against infections in gut mucosal tissue. Follicular helper T (Tfh) cells are located in germinal centers and promote IgA production via interactions with germinal center B cells. Several studies have demonstrated that some lactic acid bacteria (LAB) strains activate the host’s acquired immune system, inducing IgA secretion in the intestine. However, the precise molecular mechanisms underlying the effects of LAB on IgA production and Tfh cells are not fully resolved. *Lactobacillus paracasei* MCC1849 is a probiotic strain isolated from the intestine of a healthy adult. In this study, we investigated the effects of orally administered heat-killed MCC1849 on IgA production in the intestine and on Tfh cell induction *in vivo*. We found that orally administered MCC1849 induced antigen-specific IgA production in the small intestine, serum and lungs. We also observed that MCC1849 increased the proportion of IgA^+^ B cells and Tfh cells in Peyer’s patches (PPs). In addition, MCC1849 increased the gene expression of IL-12p40, IL-10, IL-21, STAT4 and Bcl-6 associated with Tfh cell differentiation. These results suggest that orally administered MCC1849 enhances antigen-specific IgA production and likely affects Tfh cell differentiation in PPs.

## Introduction

Host immune system responses against infections can be divided into two classes, innate and acquired responses. Immunoglobulin (Ig) A plays a major role in the mucosal defense of the host against infections as a part of acquired immunity. The primary role of mucosal IgA has been reported to be the neutralization of harmful bacteria and viruses by interfering with their motility or by inhibiting their adherence to epithelial cells [1]. Gut-associated lymphoid tissues (GALTs), such as Peyer’s patches (PPs), are important inductive sites for the initiation and generation of IgA-committed B cells [2]. Several studies have revealed that dendritic cells (DCs) in PPs induce IgA class-switch recombination (CSR) through a T cell-independent pathway by producing retinoic acid (RA), a proliferation-inducing ligand (APRIL) and B-cell activating factor (BAFF) [3]. DCs may also enhance the development and/or secretion of IgA-producing cells [4]. In addition, antigen-specific helper T cells are primed and activated by DCs to support the IgA CSR of IgM^+^ B cells to IgA^+^ B cells through the secretion of transforming growth factor-β (TGF-β), interleukin-4 (IL-4) and the CD40 ligand [5]. Antigen-specific IgA production and IgA^+^ B cell development in PPs are regulated by follicular helper T (Tfh) cells [6]. Tfh cells have recently been proposed to be another distinct subset of helper T cells that play roles in enhancing germinal center formation and regulating germinal center B cell differentiation [7]. Tfh cells are characterized by the selective expression of a variety of molecules, including the surface markers CXC chemokine receptor 5 (CXCR5), programmed death 1 (PD-1), and inducible co-stimulator (ICOS), and the production of the cytokine IL-21 [8]. IL-6 and IL-21 act via STAT3 and have been proposed to be drivers of Tfh cell differentiation [9].

Probiotic bacteria, such as lactic acid bacteria (LAB), have been suggested to be beneficial to the host and are widely used in dietary supplements, food, and infant formula. There are some reports that LAB stimulate host immune cells and protect against infection [10,11]. Some LAB strains enhance innate immunity, inducing IL-12 production by antigen-presenting cells (APCs), which activates host NK cells and promotes type 1 helper T (Th1) cell differentiation [12]. There are some reports that LAB enhance acquired immunity and induce IgA secretion in the intestine [13–15] as well as promote IgA production and increase the number of IgA^+^ B cells in mice [16][17]. Reports also show that LAB effectively enhance innate and acquired immune responses [13,18,19]. However, the precise molecular mechanisms underlying the effects of LAB on both immune systems have not been fully resolved. Recently, it was shown that IL-12, acting via the transcription factor STAT4, induced both the *Il21* and *Bcl6* genes, generating cells with features of both Tfh and Th1 cells [20]. These results led us to hypothesize that LAB with greater capacities for inducing IL-12 production may enhance Tfh cell differentiation and promote IgA secretion.

*Lactobacillus paracasei* MCC1849 is a probiotic strain that was isolated from the intestine of a healthy adult. This strain has a high capacity for inducing IL-12 production in murine splenocytes, and it has been shown that the administration of heat-killed MCC1849 enhances the antibody response against IFV vaccination in elderly over 85 years old [21]. MCC1849 may affect host acquired immune responses against infection; however, the underlying mechanism of the effects of MCC1849 are still unclear. In this study, we investigated the effects of orally administered heat-killed MCC1849 on antigen-specific IgA production in the intestine and on Tfh cell induction *in vivo*.

## Materials and methods

### Ethics statements

The animal experiments concerned with IgA production were approved by the Ethics Committee of Morinaga Milk Industry (Permit Number: 14–005, 14–039 and 14–040) and were performed in accordance with the Guide for the Care and Use of Laboratory Animals of Morinaga Milk Industry; with the Law Concerning the Protection and Control of Animals and with Standards Relating to the Care and Management of Laboratory Animals and Relief of Pain. The animal experiments concerned with influenza virus infection were approved by the Experimental Animal Research Committee for Ethics and Animal Experimentation, Nihon Bioresearch Inc. (Project No.076132).The experiments were conducted in accordance with the Guidelines for Management and Welfare of Experimental Animals (Hashima Laboratory, Nihon Bioresearch Inc., October 2, 2007; modified on August 27, 2010).

### Mice

Male SPF BALB/c mice were obtained from SLC Japan Inc. (Shizuoka, Japan). Mice were housed in plastic cages and used when 7 weeks old. Mice were provided a purified diet (AIN93-G; Funabashi Farm, Chiba, Japan) and water ad libitum throughout the experimental period. Mice were sacrificed under isoflurane anesthesia by experts, and all efforts were made to minimize suffering.

### Microorganisms

*Lactobacillus paracasei* MCC1849 and type strains of *Lactobacillus*, including *Lactobacillus delbrueckii* subsp. *lactis* (JCM1248T), *Lactobacillus casei* (JCM1134), *Lactobacillus gallinarum* (ATCC33199), *Lactobacillus plantarum* (ATCC14917), *Lactobacillus reuteri* (JCM1112), *Lactobacillus crispatus* (JCM1185), *Lactobacillus johnsonii* (ATCC33200), *Lactobacillus paracasei* subsp. *paracasei* (JCM8130), *Lactobacillus helveticus* (JCM1120), and *Lactobacillus bulgaricus* (ATCC11842), were either obtained from stock cultures maintained in the Morinaga Culture Collection (MCC; Morinaga Milk Industry Co., Ltd., Zama, Japan) or purchased from the American Type Culture Collection (ATCC; Manassas, VA, USA) or the Japan Collection of Microorganisms (JCM; Wako, Japan). These organisms were cultured for 16 h at 37 °C in Lactobacilli-MRS broth (DIFCO, Mich., USA), collected via centrifugation, washed twice with phosphate-buffered saline (PBS), and then washed twice with sterile distilled water. The organisms were suspended in distilled water and were killed by heating them at 100 °C for 30 min. A portion of each heated suspension was lyophilized to measure the dry weight of the bacterial cells in the suspension. The concentration of the heat-killed *Lactobacillus* in each suspension was adjusted to 10 mg/ml (dry weight) with distilled water.

### Cell cultures

Splenocytes were obtained from mice euthanized via cervical dislocation and treated with a Tris-buffered NH_4_Cl solution to deplete erythrocytes. Splenocytes were prepared as a single-cell suspension (2.5 × 10^6^ cells/ml) and cultured in Roswell Park Memorial Institute (RPMI) 1640 medium (Gibco, Carlsbad, CA, USA) supplemented with 10% heat-inactivated fetal bovine serum (FBS), 100 U/ml penicillin, 100 μg/ml streptomycin, and 50 μM 2-mercaptoethanol with or without heat-killed *Lactobacillus* (10 μg/ml) in a 96-well culture plate at 37 °C in 5% CO2. Culture supernatants were collected on day 2 and kept at -80 °C until analysis.

### Influenza virus (IFV) infection

IFV infection was evaluated in accordance with the methods of Iwabuchi *et al* [12]. Mice were orally administered 1 mg/0.2 ml/mouse of lyophilized MCC1849 daily beginning 2 weeks before IFV infection and continuing until one day before sacrifice (MCC1849 group; n = 10). As a control, mice were given an equal volume of saline (Control group; n = 10). All mice were infected intranasally with 50 μl of saline containing 5 × 10^6^ pfu of IFV A/PR/8/34(H1N1) [12]. Following infection, mice were monitored daily for symptoms of infection based on their eyes (extent of lid closure and eyelid reflex) fur, behavior (extent of locomotor activity), and breathing (extent of irregular respiration). Each condition was scored on a scale from 0 to 4 as follows: 0, normal; 1, mild; 2, moderate; 3, severe; and 4, death. Symptom scores for each mouse were estimated from the average score for these conditions. Six days after infection, mice were sacrificed, and their lungs were extracted. Viral titers of the lung homogenate were determined using a plaque assay. No animals died before meeting the humane endpoint criteria.

### Examination of IgA and oral ovalbumin (OVA) immunization

Mice were divided into two groups, control and MCC1849, with similar mean body weights and total fecal IgA levels. The MCC1849 group was fed approximately 0.5 g of the AIN-93G diet that had 40 mg of heat-killed MCC1849 added to it. The control group was fed 0.5 g of the AIN-93G diet separately from their usual diet. Mice were fed for 5 weeks with or without MCC1849 once a day. OVA-immunized mice were orally immunized with 1 mg of OVA (grade V; Sigma, St. Louis, MO, U.S.A.) and 10 μg of cholera toxin (Sigma, St Louis, MO) on days 14, 21, 28. Mice were sacrificed under isoflurane anesthesia on day 35 and sera, small intestines, colons, lungs and mesenteric lymph nodes (MLNs) were collected from the mice.

### Preparation of tissues

Small-intestine lavage fluid was collected by washing out intestines with 4 ml of PBS containing a protease inhibitor cocktail (Roche Diagnostics, Rotkreuz, Switzerland). The fluid was centrifuged at 9,200 × *g* for 15 min at 4 °C, and the supernatant was then collected. PPs were collected from the small intestines. Isolated small intestines and colons were opened longitudinally to remove their contents and then washed with PBS. Intestinal tissues and lungs were added to 1 ml of PBS containing a protease inhibitor cocktail (Roche), 40 mM HEPES, 1% Triton and 10% glycerol. These tissues were homogenized and centrifuged at 9,200 × *g* for 15 min at 4 °C, and the supernatant was then collected. Colon contents were collected and suspended in PBS containing 50 mM EDTA and 0.1 mg/ml of a trypsin inhibitor. This suspension was vigorously vortexed with glass beads and centrifuged at 15,000 × *g* for 15 min at 4 °C. Suspensions were centrifuged again at 10,000 × *g* for 15 min, and the supernatant was used for the detection of total IgA and OVA-specific IgA using a mouse IgA ELISA (Bethyl Laboratory, TX, USA). Total protein levels were measured using BCA Protein Assay Kits (PIERCE, IL).

### Isolation of lymphocytes

Both PP and MLN cells were digested with collagenase Type I (Sigma, St. Louis, MO) and single-cell suspensions were prepared. For the isolation of lamina propria (LP) cells, small intestines were removed after excluding PPs and then cut into 1-cm pieces and opened up. The small intestines were washed with 40 ml of Hank’s balanced salt solution (HBSS) (Gibco) supplemented with 5% FBS and 2.5 mM EDTA in a 50-ml Erlenmeyer flask and incubated at 37 °C for 30 min with shaking at 150 rpm. The tissues were incubated with RPMI 1640 medium containing 10% FBS, 100 μg/ml penicillin and 100 U/ml streptomycin (Invitrogen) supplemented with 0.25 mg/ml collagenase and 10 μg/ml DNase I (Roche) at 37 °C for 30 min with stirring. Cells were collected via centrifugation and were suspended in 40% Percoll solution (GE Healthcare, UK). This suspension was overlaid on 70% Percoll solution and centrifuged at 1900 × *g* for 20 min. Cells in the boundary between 40% and 70% Percoll solutions were collected. The isolated LP cells were washed for subsequent use.

### Flow cytometric analysis

PP cells, MLN cells and LP cells (5 × 10^5^ cells) were washed with 1 ml of FACS buffer (PBS containing 1% FBS and 0.1% sodium azide) and centrifuged at 400 × *g* for 5 min at 4 °C. The supernatant was aspirated, and suspensions were treated with CD16/32(eBioscience) for 20 min at 4 °C. Thereafter, the cells were stained with the following monoclonal antibodies (mAbs): FITC-labeled anti-mouse CD45R/B220 (RA3-6B2, eBioscience), FITC-labeled anti-mouse CD4 (GK1.5, eBioscience), PE-labeled anti-mouse IgA (11-44-2, eBioscience), PE-labeled anti-mouse CD185(CXCR5) (SPRCL5, eBioscience), PerCP-Cyanine5.5-labeled anti-mouse CD45 (30-F11, eBioscience), APC-labeled anti-mouse CD279(PD-1) (J43, eBioscience), and APC-labeled anti-mouse CD45R/B220 (RA3-6B2, eBioscience). After a thorough mixing, the cells were incubated for 30 min in the dark at 4 °C, washed with 1 ml of FACS buffer and centrifuged at 400 × *g* for 5 min. The supernatant was completely aspirated, and the cells were resuspended in 500 μl of FACS buffer. Cell analyses were carried out using a FACSCanto system (BD Biosciences), with the data obtained using FlowJo ver. 7.6 (Tree Star, Ashland, OR, USA)

### Cytokine and IgA measurements

Total IgA levels were assessed using an ELISA kit (Bethyl Laboratory, TX, USA) according to the manufacturer’s instructions. OVA-specific IgA levels were evaluated using an ELISA in accordance with the methods of Sato *et al* [22]. OVA was suspended in 0.05 M carbonate-bicarbonate buffer (pH 9.6). Instead of the primary antibody in the mouse IgA ELISA kit, this solution was used to coat the ELISA plate at 20 mg/ml. Subsequently, the kit was used according to the manufacturer’s instructions. Standard serum levels for the OVA-specific immunoglobulin assays were obtained from the sera of mice immunized via three intraperitoneal injections of 1 mg OVA mixed in 0.1 ml of Freund's Complete Adjuvant (Difco, USA) at 2-week intervals. The OVA-specific IgA titers of the standard serum samples were arbitrary set at 10000 units/ml, and the titers of experimental samples are expressed relative to this standard serum value [22]. Concentrations in the test samples were calculated in arbitrary units relative to a standard curve generated from pooled plasma samples. Mouse IL-12p70 in the culture supernatants was quantified using ELISA kits (R&D Systems, Minneapolis, MN, USA) in accordance with the manufacturer’s instructions.

### RNA extraction and quantitative real time-PCR analysis

Total RNA from PP and small intestine samples was extracted with QIAzol reagent and isolated using an RNeasy Mini Kit (QIAGEN, Hilden, Germany) according to the manufacturer’s instructions. cDNA was synthesized using a PrimeScript RT Reagent Kit (TaKaRa) according to the manufacturer’s instructions. Quantitative real-time RT-PCR was performed using SYBR Premix Ex Taq (TaKaRa) in an ABI 7500 RT-PCR machine (Applied Biosystems). Real-time PCR analyses were run at 95 °C for 30 s and then for 40 cycles of 95 °C for 3 s and 60 °C 30 s. Expression levels were normalized to the housekeeping gene GAPDH, which was used as an internal control. The sequences of the primers were as follows: IL-6, 5’-TGGAGTCACAGAAGGAGTGGCTAAG-3’ and 5’-TCTGACCACAGTGAGGAATGTCAAC-3’; IL-10, 5’-CCAAGCCTTATCGGAAATGA-3’ and 5’-TTTTCACAGGGGAGAAATCG-3’; IL-12p40, 5’- TGGTTTGCCATCGTTTTGCTG -3’ and 5’- ACAGGTGAGGTTCACTGTTTCT -3’; IL-21, 5’-CGCCTCCTGATTAGACTTCG-3’ and 5’-TGTTTCTTTCCTCCCCTCCT-3’; IFN-γ, 5’-ACTGGCAAAAGGATGGTGAC-3’ and 5’-TGAGCTCATTGAATGCTTGG-3’; STAT3, 5’-GACCCGCCAACAAATTAAGA-3’ and 5’-TCGTGGTAAACTGGACACCA-3’; STAT4, 5’-CATCCCTGAAAACCCTCTGA-3’ and 5’-GACATGGGGAGAAGGTCTGA-3’; Bcl-6, 5’-CCTGAGGGAAGGCAATATCA-3’ and 5’-CGGCTGTTCAGGAACTCTTC-3’; pIgR, 5’-GCTCCAAAGTGCTGTTCTCC-3’ and 5’-TTGCTGTGTGTCTGGAGAGG-3’; and GAPDH, 5’-TGTGTCCGTCGTGGATCTGA-3’ and 5’-TTGCTGTTGAAGTCGCAGGAG-3’.

### Statistics

Experimental data are expressed as the mean ± standard deviations. Statistical analyses were conducted using Student’s t-tests with effect size reported as Cohen’s d (d)P values < 0.05 were considered to be statistically significant.

## Results

### MCC1849 induces high levels of IL-12 production and protects against IFV infection

**Fig 1:**
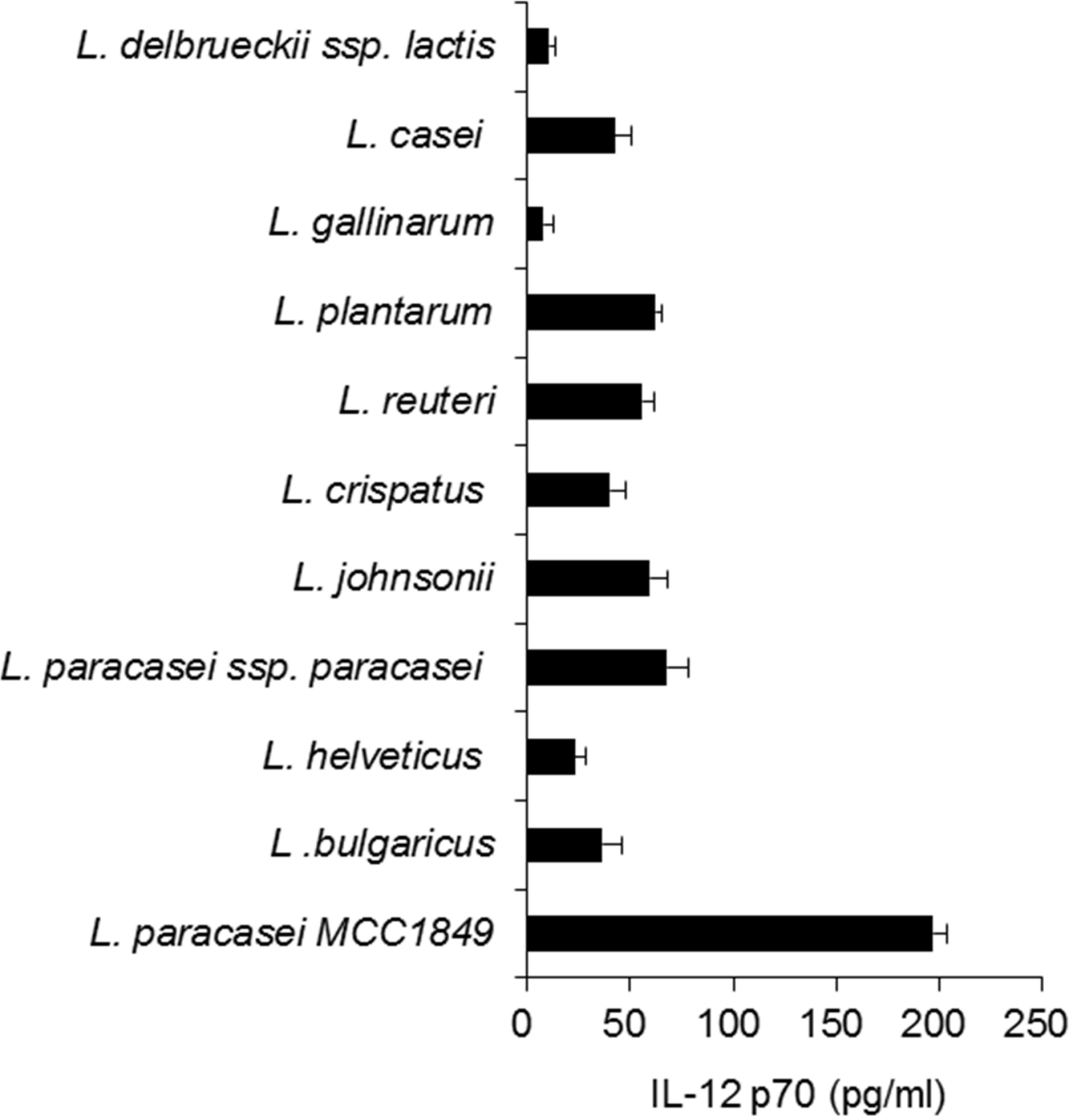
Effects of *Lactobacillus paracasei* MCC1849 on IL-12 production. Murine splenocytes were cultured with various heat-killed *Lactobacillus* strains (10 μg/ml) for 2 days. Data shown are the mean ± SD of the levels of IL-12p70 which are the representative of three independent experiments.

We compared effects on IL-12 induction of some *Lactobacillus* strains (Fig 1). The *L*. *paracasei* strain MCC1849 induced high levels of IL-12 when compared with the effects of other *Lactobacillus* type strains. The oral administration of MCC1849 to mice from 2 weeks before IFV infection resulted in significantly lowered symptom scores from 6 days after the infection and the significant inhibition of viral proliferation in the lungs when compared with the response in the control group (S1 Fig).

### MCC1849 induces IgA^+^ cells in intestinal tissue and promote IgA secretion in the small intestine

**Fig 2:**
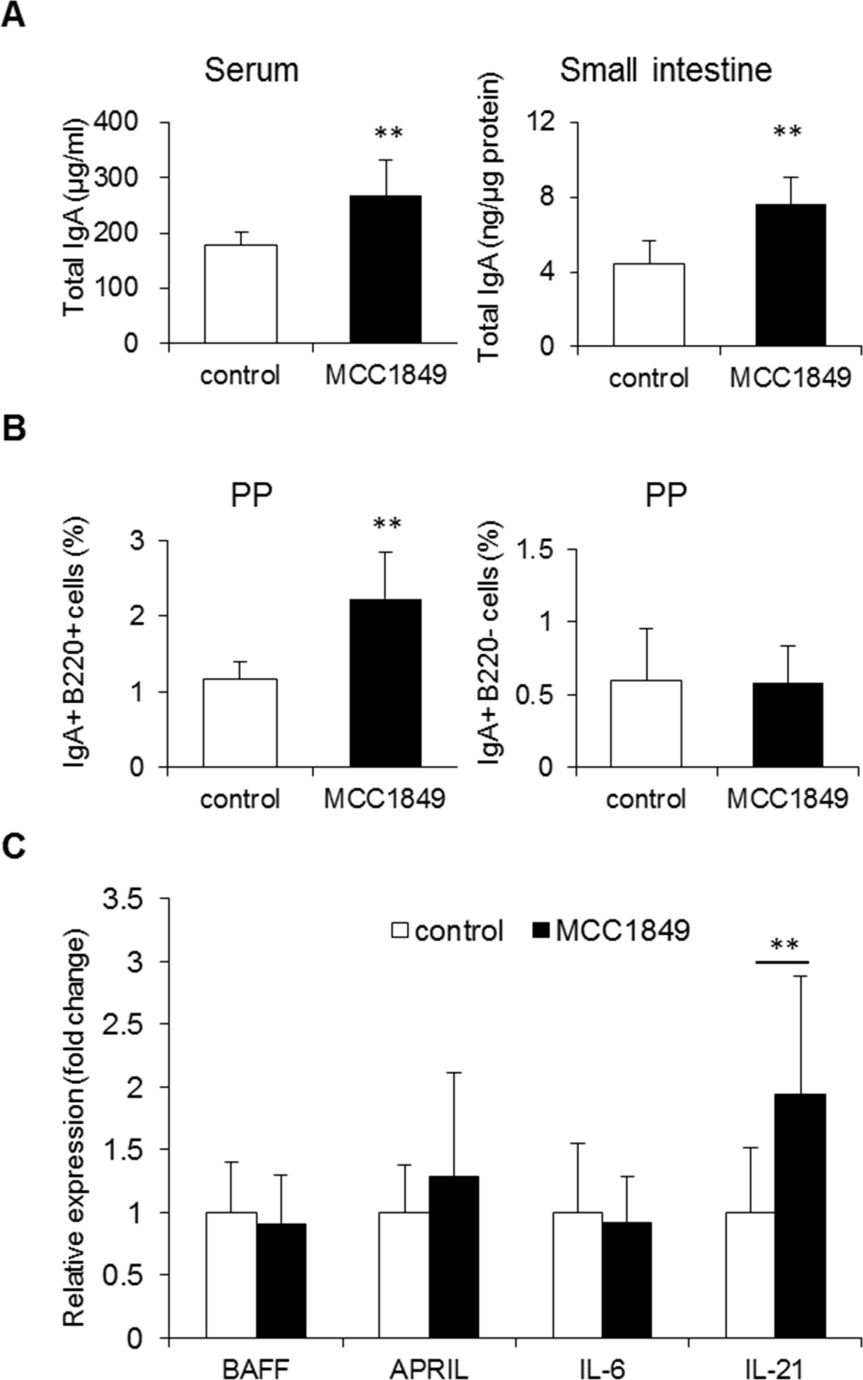
Effects of *Lactobacillus paracasei* MCC1849 on total IgA production in mice. (A) Mice were treated with or without MCC1849 for 5 weeks. Total IgA concentrations in homogenized small intestine and serum samples were determined via ELISA. (B) The proportions of IgA^+^ B220^+^ cells and IgA^+^ plasmablasts (IgA^+^ B220^-^ cells) in PPs were analyzed via FCM. (C) Gene expression related to the differentiation of IgA^+^ cells was measured via real-time RT-PCR analysis. The level of gene expression was normalized to that of GAPDH mRNA expression in the control group. Data are shown as the mean ± SD. N = 16. *p<0.05, **p<0.01 compared to the control.

To investigate the effects of MCC1849 on IgA production, we evaluated IgA induction in the small intestines and sera of mice orally administered MCC1849 for 5 weeks. Total IgA levels in small intestine and serum samples were significantly higher in the MCC1849-fed group than in the control group (Fig 2A). The proportion of IgA^+^B220^+^ cells in PPs was approximately two-fold higher in the MCC1849-fed group than in the control group, but the proportion of IgA^+^ plasmablasts (IgA^+^ B220^-^ cells) was not different between the two groups (Fig 2B). IL-21 gene expression in PPs was increased in the MCC1849-fed group relative to that in the control group, but BAFF, APRIL and IL-6 expression were not different (Fig 2C). Effect size (Cohen's d) for the resulting differences between control group and MCC1849-fed group were calculated as an additional quality criterion. Effect sizes between control group and MCC1849-fed group were d>0.8, which were considered sufficiently as large.

### MCC1849 induces antigen-specific IgA production in OVA-immunized mice

**Fig 3:**
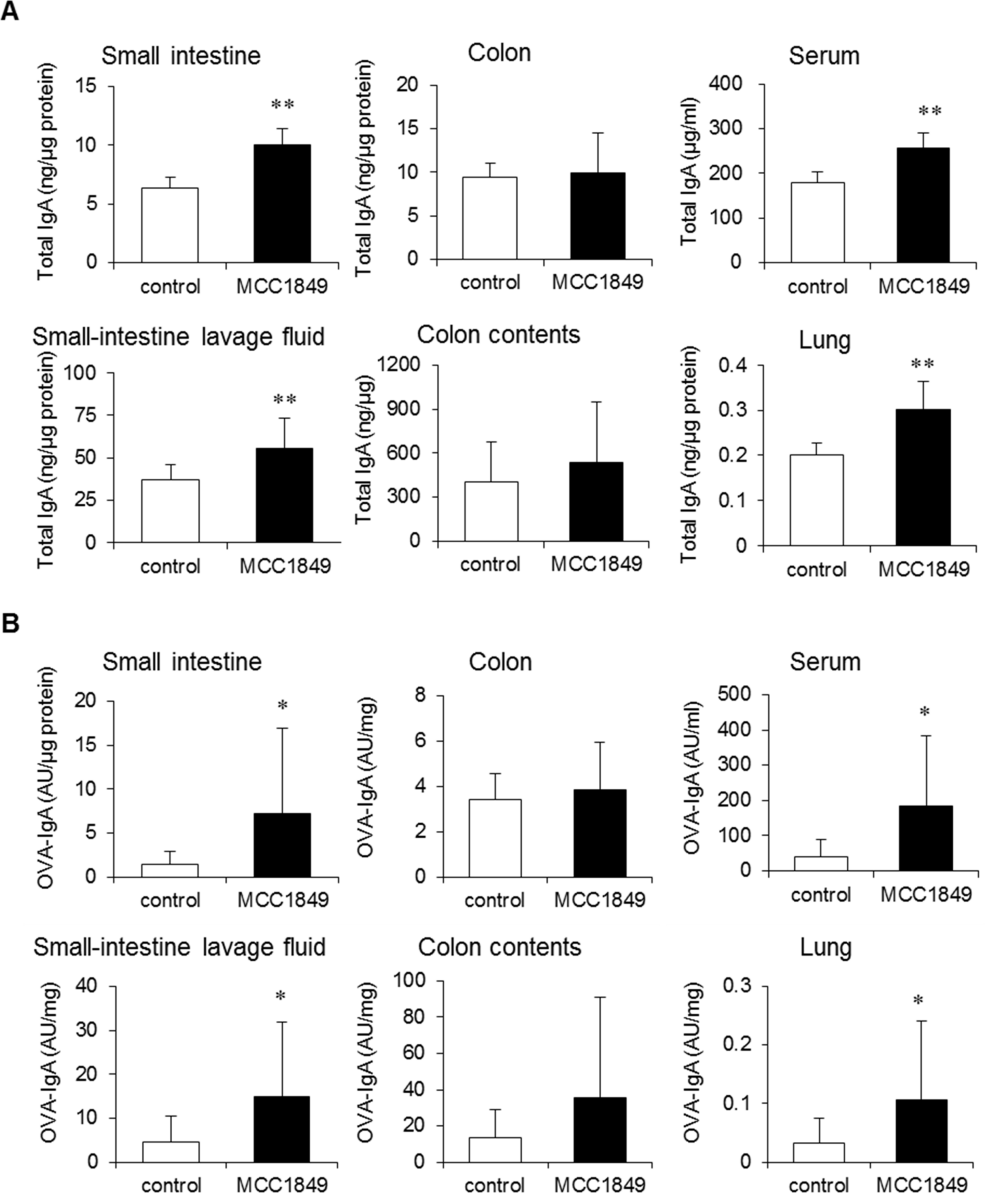
Effects of *Lactobacillus paracasei* MCC1849 on total IgA and OVA-specific IgA production in OVA-immunized mice. (A, B) Mice were treated with or without MCC1849 for 5 weeks. All mice were orally immunized on days 14, 21, and 28 with OVA and cholera toxin. On day 35, mice were euthanized and dissected. Data show the total IgA and OVA-specific IgA concentrations in homogenized small intestine, small-intestine lavage fluid, homogenized colon, colon contents, serum and lung samples. AU: arbitrary unit. Data are shown as the mean ± SD. N = 14. Data are representative three independent experiments. *p<0.05, **p<0.01 compared to the control.

We evaluated antigen-specific IgA production in each mouse group following oral OVA immunizations. Both total IgA and OVA-specific IgA levels in the lavage fluid from small intestines, homogenized small intestines, sera and lungs were significantly increased in the MCC1849-fed group compared with the levels in the control group. Effect sizes between control group and MCC1849-fed group were d>0.8. However, total IgA and OVA-specific IgA levels in the colon contents and homogenized colon samples were not significantly different between the groups (Fig 3).

**Fig 4:**
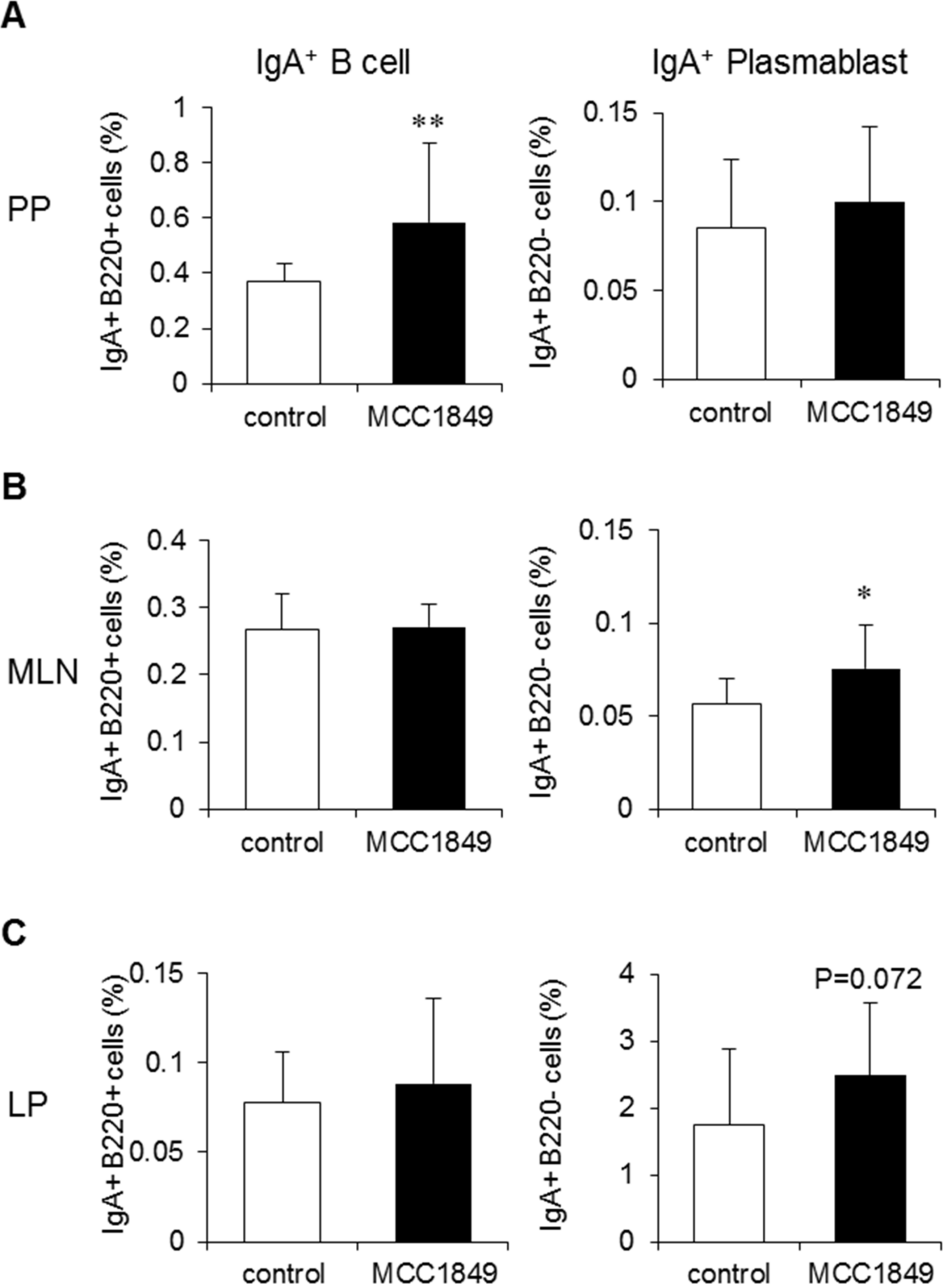
Effects of *Lactobacillus paracasei* MCC1849 on the population of IgA-related cells in the intestinal tissues of OVA-immunized mice. (A, B and C) The proportion of IgA^+^ B220^+^ cells and IgA^+^ plasmablasts (IgA^+^ B220^-^ cells) in PP, MLN and LP cell samples were analyzed via FCM. Data are shown as the mean ± SD. N = 14. Data are representative two independent experiments. *p<0.05, **p<0.01 compared to the control.

We investigated the proportion of IgA^+^ B220^+^ cells and IgA^+^ plasmablasts (IgA^+^ B220^-^ cells) in PP, MLN and LP cell samples. The proportion of IgA^+^ B220^+^ cells in PP cell samples was significantly increased in the MCC1849-fed group compared with the proportion in the control group (d = 1.02). The proportion of IgA^+^ plasmablasts was increased in MLN cell samples and tended to be increased in LP cell samples (P = 0.07) in the MCC1849-fed group compared with the proportions in the control group (d = 0.99 and 0.66, respectively) (Fig 4).

### MCC1849 induces Tfh cells and affects cytokine gene expression in PPs

**Fig 5:**
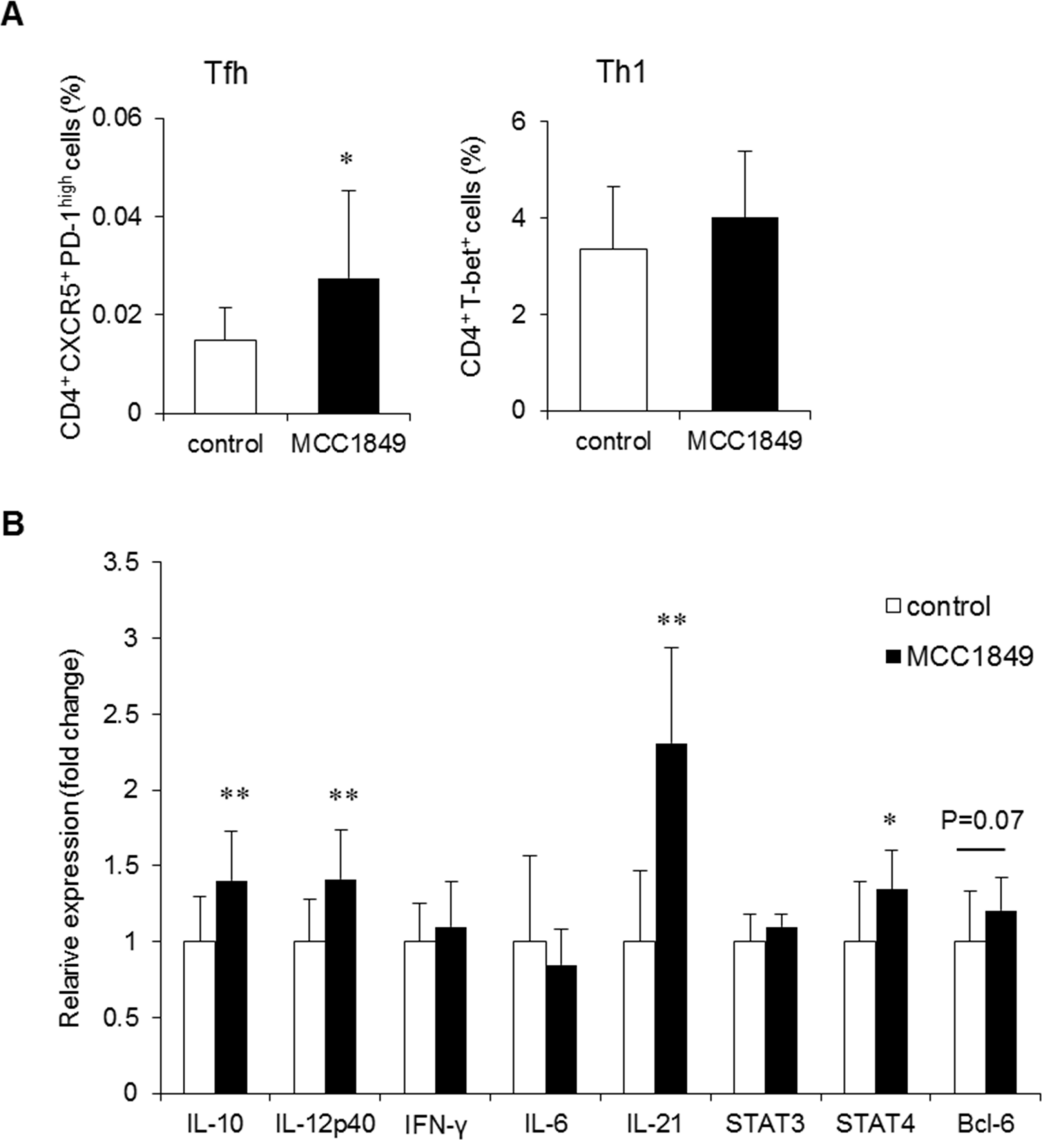
Effects of *Lactobacillus paracasei* MCC1849 on the population of T cells and gene expression in PP cells in OVA-immunized mice. (A) The proportion of Tfh cells (CD4^+^ CXCR5^+^ PD-1^high^ cells) and Th1 cells (CD4^+^ T-bet^+^ cells) in PPs were analyzed via FCM. (B) Gene expression related to T cell differentiation was measured via real-time RT-PCR analysis. The level of gene expression was normalized to that of GAPDH mRNA expression in the control group. Data are shown as the mean ± SD. n = 14. Data are representative two independent experiments. *p<0.05, **p<0.01 compared to the control.

We investigated the induction of Tfh cells required for IgA^+^ B cell differentiation in PPs. The proportion of Tfh cells (CD4^+^ CXCR5^+^ PD-1^high^ cells) in PP cell samples was significantly increased in the MCC1849-fed group compared with the proportion in the control group (d = 0.94), but the proportion of Th1 cells (CD4^+^ T-bet^+^ cells) was not different between the two groups (Fig 5A). IL-10, IL-12p40, IL-21 and STAT4 gene expression were significantly increased and Bcl-6 tended to be increased (P = 0.07) in PPs from the MCC1849-fed group compared with expression in the control group, but IFN-γ, IL-6 and STAT3 expression levels were not different (Fig 5B).

## Discussion

LAB species have been reported to modulate host immune responses [23], and there are many reports that some probiotic LAB induce IL-12 production, affecting intestinal DCs and enhancing host innate immunity [24]. We have previously reported that heat-killed MCC1849 affects host acquired immunity and increases the antibody response against IFV vaccination in elderly over 85 years old [21]. The capacity of MCC1849 for IL-12 induction is considerably greater than that of other *Lactobacillus* type strains (Fig 1A). In the present study, we showed that the administration of MCC1849 induced antigen-specific IgA production and increased the proportion of Tfh cells in PPs (Figs 2–5).

We found that MCC1849 induced a statistically significant increase in total IgA production in the small intestine and serum (Fig 2A). In addition, the proportion of IgA^+^ B cells in PPs was significantly higher than in the control. It has been reported that some LAB induce APRIL and BAFF, which act to induce the IgM-to-IgA CSR via a T cell-independent pathway. However, we found no change in APRIL and BAFF gene expression in PPs following the administration of MCC1849 (Fig 2C). MCC1849 may induce the IgM-to-IgA CSR in PPs and IgA production in the small intestine through a mechanism other than a T cell-independent pathway. It is known that IL-21 is secreted from Tfh cells in PPs and induces an antigen-specific T cell-dependent IgA CSR in B cells [25]. In our study, we observed that IL-21 gene expression was significantly higher in PP cells from MCC1849-fed mice (Fig 2C). Antigen-specific Tfh cells are necessary for B cells to produce antigen-specific IgA in PP cells [26], and it has been reported that antigen-specific IgA plays an important role against mucosal infections and acts to neutralize and eliminate exogenous antigens [27]. We confirmed that orally administered MCC1849 induces antigen-specific IgA production and affects Tfh cell populations in PPs. In the experiments using OVA-immunized mice, orally administered MCC1849 induced OVA-specific IgA production and increased the proportion of Tfh (CD4^+^ CXCR5^+^ PD-1^high^) cells in PPs (Figs 3B and 5A). Recent findings have shown that IL-12 induces the development of transitional cells from naïve T cells that differentiate both to Th1 and Tfh cells [20]. The cytokines IL-12 and IFN-γ, acting via STAT4 and STAT1, are important for specification and cell fate commitment of Th1 cells [28], whereas IL-6 and IL-21, acting via STAT3, have been proposed to be drivers of Tfh cell differentiation [29]. In orally immunized mice, cytokine gene expression in PP cells was changed in response to MCC1849 feeding. IL-12, IL-21, STAT4 and Bcl-6 gene expression levels were significantly higher than in the control, whereas IL-6, IFN-γ and STAT3 expression did not increase. It has been reported Bcl-6 expression and IL-21 production induce polarization to Tfh cells and that T-bet expression and IFN-γ production induce polarization to Th1 cells from transitional cells [20]. In our study, IFN-γ expression and the proportion of Th1 cells in PPs were not changed following MCC1849 feeding; however, IL-21 expression was significantly higher than in the control. These results suggest that MCC1849, which results in elevated IL-12 production, may first induce transitional cell development from naïve cells and then drive Tfh cell differentiation from transitional cells via the induction of IL-21 and Bcl-6.

The oral administration of MCC1849 induced IgA^+^ B cells in PPs and tended to increase IgA^+^ plasmablasts in the LP, although it did not change the frequency of IgA^+^ plasmablasts in PPs (Fig 4A). IL-6 or IL-10 may induce the differentiation of IgA-producing plasmablasts in IgA^+^ B cell populations [30]. We confirmed that IL-10 gene expression is increased in PPs from MCC1849-fed mice; however, IL-6 gene expression did not change compared with expression in the control. These results suggest that MCC1849 induces IL-10 production in PPs and that IL-10 may promote the differentiation of IgA^+^ B cells into IgA^+^ plasmablasts, which home to the LP and secrete IgA [31]. However, the effects of MCC1849 on gut-homing receptors are not clear, and further investigation is needed. In addition, it is known that IgA is secreted through the pIgR receptor to the gut lumen [32]. pIgR gene expression levels in the small intestine were also significantly increased by MCC1849 (S2 Fig). These results suggest that orally administered MCC1849 enhances IgA secretion in the small intestine in addition to inducing IgA^+^ B cells by stimulating APCs in PPs.

Orally administered MCC1849 did not clearly induce total IgA and OVA-specific IgA production in the colon and its contents. It has been reported that the mechanism responsible for the induction of IgA production by commensal bacteria differs for the small intestine and the large intestine [33]. A previous study using gnotobiotic mice for *Clostridium* showed that *Clostridium* did not induce IgA production in the small intestine but did in the large intestine [34]. In large-intestine tissues, IgA production and IgA^+^ cell numbers in *Bacteroides* mono-associated mice were significantly higher than in germ-free mice and *Lactobacillus* mono-associated mice [33]. These reports suggest that particular microorganisms, such us *Clostridium* and *B*. *acidifaciens*, promote IgA production in the large intestine. There are some reports that Lactobacillus strains induce higher levels of IgA in the small intestine [14,15]. These reports may suggest that commensal bacteria responsible for the induction of IgA are different between the small and large intestines, and it is possible that *Lactobacillus* strains induce IgA production mainly in the small intestine and not in the large intestine.

MCC1849 increased IgA levels in not only the small intestine but also in the serum and lungs. These results suggest that MCC1849 may modulate not only gut mucosal immunity but also respiratory and systemic immunity. It has been reported that the microbiota regulates immune defense against IFV infection [35]. In our present study, we confirmed the effects of orally administered MCC1849 against influenza infections in mice. We observed that MCC1849 suppressed the viral titers in the lungs, improved the histopathological findings, and induced increases in IgA levels in the lungs. These results suggest that MCC1849 may affect the mucosal immune response against infection in the respiratory tract mucosa. Further studies are needed to investigate the mechanism underlying the protective effects of MCC1849 against virus infection.

In this study, we investigated the effect of orally administrated MCC1849 on immune stimulation focusing on IgA production. It is interesting to consider the effect of MCC1849 on IgA selection and immune tolerance. It has been reported that some probiotic strains could induce oral tolerance through affecting intestinal DC cells and regulatory T cells[36].It is known that follicular regulatory T (Tfr) cells in the germinal center of PPs are important role for regulation of IgA selection[37]. Therefore, MCC1849 may induce IgA production through not only Tfh cells but also Tfr cells in PPs, but further investigation is needed. In addition, administration of antigens into the anterior chamber (AC) of the eye induces a form of antigen-specific immune tolerance termed anterior chamber-associated immune deviation (ACAID). ACAID antigen presentation via APCs and B cells leads to the generation of antigen-specific T regulatory cells[38]. It may be possible that eye-injected MCC1849 and antigen may induce antigen-specific regulatory T cells and subsequent immune tolerance.

In conclusion, we demonstrated that orally administered heat-killed MCC1849 enhances antigen-specific IgA production in intestinal tissues, suggesting that MCC1849 has the potential to enhance acquired immune responses. Furthermore, our results provide the first evidence of the LAB-induced differentiation of Tfh cells in the intestinal immune system. Further studies are now underway to determine the precise mechanism underlying Tfh induction by MCC1849.

## Supporting information

S1 Fig Effects of *Lactobacillus paracasei* MCC1849 against IFV infection. (A) Mice were orally administered lyophilized MCC1849 daily from 2 weeks before IFV infection to one day before sacrifice (MCC1849 group). As a control, mice were given an equal volume of saline (Control group). All mice were infected intranasally with 50 μl of saline containing 5 × 10^6^ pfu of the IFV. Following infection, mice were monitored daily for infection symptoms. (B) Virus titers of the lung on day 6.
(TIF)

S2 Fig Effects of *Lactobacillus paracasei* MCC1849 on the gene expression of IgA secretion in intestinal tissues in OVA-immunized mice. Gene expression of polymeric Immunoglobulin receptor (pIgR) in small intestine was measured by real-time RT-PCR analysis. The level of gene expression was normalized to that of GAPDH mRNA expression in control group. Data are shown as mean ± SD. *p<0.05, **p<0.01, paired t-test.
(TIF)
