# Supplementary figures and images for: Orally administered heat-killed Lactobacillus paracasei MCC1849 enhances antigen-specific IgA secretion and induces follicular helper T cells in mice
Source: PLoS One. 2018 Jun 13;13(6):e0199018. doi: 10.1371/journal.pone.0199018 (PMC5999281; doi:10.1371/journal.pone.0199018)

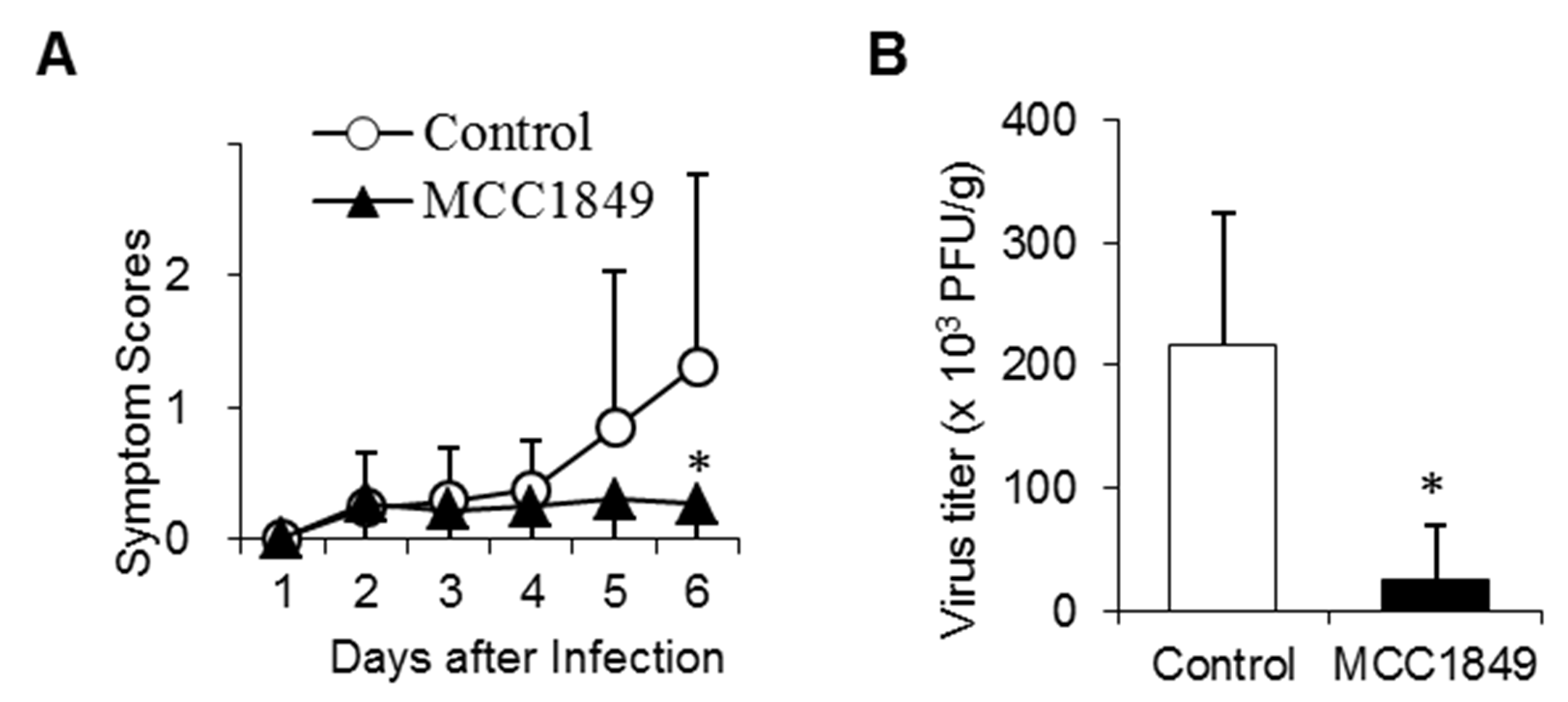

Supplement: S1 Fig — (A) Mice were orally administered lyophilized MCC1849 daily from 2 weeks before IFV infection to one day before sacrifice (MCC1849 group). As a control, mice were given an equal volume of saline (Control group). All mice were infected intranasally with 50 μl of saline containing 5 × 10^6^ pfu of the IFV. Following infection, mice were monitored daily for infection symptoms. (B) Virus titers of the lung on day 6. (TIF) [file pone.0199018.s001.tif]

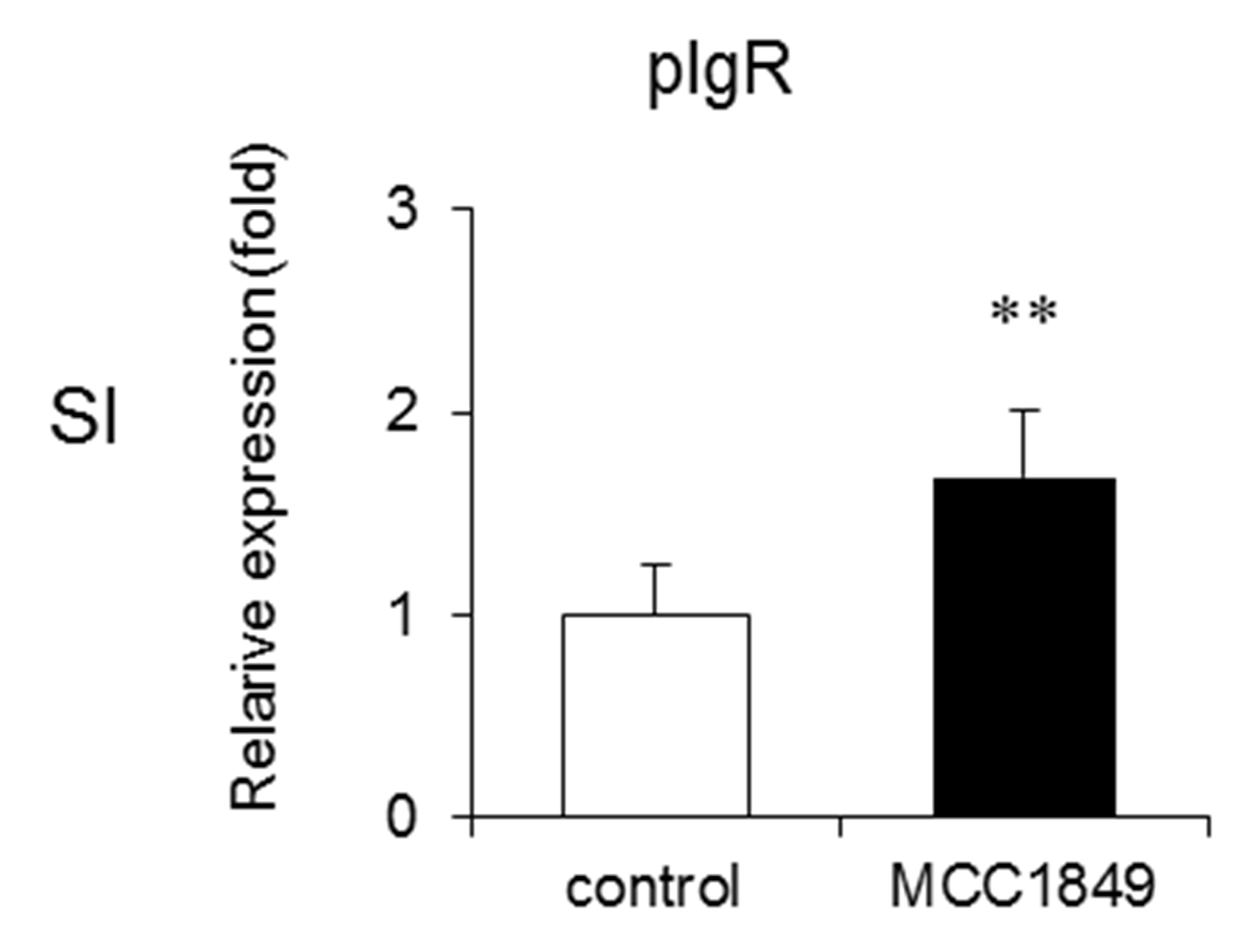

Supplement: S2 Fig — Gene expression of polymeric Immunoglobulin receptor (pIgR) in small intestine was measured by real-time RT-PCR analysis. The level of gene expression was normalized to that of GAPDH mRNA expression in control group. Data are shown as mean ± SD. *p<0.05, **p<0.01, paired t-test. (TIF) [file pone.0199018.s002.tif]
